# Recurrent pannus formation causing prosthetic aortic valve dysfunction: Is excision without valve re-replacement applicable?

**DOI:** 10.1186/1749-8090-7-62

**Published:** 2012-06-29

**Authors:** Ahmad K Darwazah

**Affiliations:** 1 Department of Cardiac Surgery, Makassed and Ramallah Hospital mount of Olives, Jerusalem, 91194, Israel

**Keywords:** Prosthetic valves dysfunction, Prosthetic aortic valve, Pannus formation, Recurrent pannus, Valve re-replacement, Surgical excision

## Abstract

Prosthetic valve dysfunction at aortic position is commonly caused by pannus formation. The exact etiology is not known. It arises from ventricular aspect of the prosthesis encroaching its leaflets causing stenosis or it may remain localized causing left ventricular outflow tract obstruction without affecting valve function.

The difference in location entails different approaches in management. Such a pathology requires surgical excision of the pannus with or without valve re-replacement.

A recurrent pannus was observed in a female patient who needed repeated surgical intervention to excise a localized pannus without re-replacement of a well functioning prosthetic valve.

Management of our case presents several questions, whether recurrence of pannus is caused by sparing the prosthetic valve, is it simply an exaggeration of an inflammatory healing process in certain individuals or is it ideal to re-replace the valve despite a well preserved function.

## Commentary

The improvement of durability and hemodynamics of currently available prosthetic valves reduced the incidence of structural valve failure to a minimum [1]. However, prosthetic valve dysfunction caused by either pannus formation or thrombosis or both is still seen in clinical practice.

Prosthetic valve dysfunction at aortic position is commonly caused by pannus formation which is an uncommon, but serious complication. Its incidence varies between 1.8% in tilting disc to 0.73% in bileaflet valves [2]. All types of available prosthetic valves can be affected by pannus formation.

The exact etiology of pannus formation is not known. Multifactors are involved in its formation. Basically, pannus represent a bioreaction to prosthetic valves associated with coexisting factors such as surgical technique, thrombus organization from inadequate anticoagulation, infection and wall shear stress [3,4].

Recent study by Teshima and colleagues [4] found that patients with prosthetic valve dysfunction secondary to pannus are associated with significant increase in the level of transforming growth factor beta(TGF-B1). This cytokine is essential for regulation of cell growth, differentiation, and matrix production. Thus, the increase production of these cytokines is implicated in the formation of pannus by inducing exaggerated healing, fibrosis and scar tissue formation.

Pannus usually originates in the neointima of the periannular tissue [5]. Histologically, it is mainly formed of collagen and elastic fibrous tissue accompanied by endothelial cells, chronic inflammatory cell infiltration and myofibroblasts [5].

In most of the previously reported cases, the formation of pannus arose from the left ventricular aspect of the prosthetic valve. In rare cases they appear to grow from the aortic side [6].

The effect of pannus formation on hemodynamics depends on the extent and site of fibrous tissue. Pannus arising from the left ventricular aspect may extend to the orifice and hinges of the prosthetic valve causing restriction of either or both leaflets causing severe narrowing of inflow orifice [7]. Severe stenosis may occur due to obstruction and narrowing of left ventricular outflow tract by a circumscribed pannus without restricting leaflet motion [1,7].

In rare cases, the formation of pannus can lead to intermittent regurgitation caused by impairment of diastolic motion of one of the prosthetic leaflets [1].

The effect of pannus on leaflet motion in some cases does not show a consistent pattern. It may cause an alternating normal movement with half closure of valve leaflets. Such an effect can produce normal hemodynamics alternating with low-cardiac output state [8]. Pannus arising from the aortic aspect of prosthetic valve usually causes complete obstruction with immobility of leaflets [6].

Recently in December (2011), a female patient aged 42 years was transferred for the 4^th^ time for open heart surgery.

She presented with severe dyspnea and limitation of activity. Transthoracic echocardiography (TTE) revealed the presence of a dysfunctional prosthetic aortic valve with stenosis across the prosthesis with maximum gradient of 75 mmHg associated with impaired left ventricular function (EF 0.30).

Inquiring about her past medical history, the patient had her first cardiac operation in June (1999) during which mitral and aortic valves were replaced by Carbomedics valve size 27 mm and 21 mm respectively.

In October (2000), the patient presented with acute heart failure secondary to dysfunctional prosthetic mitral valve. She underwent an emergency re-replacement of the mitral valve by using a St. Jude Medical valve 27 mm. During operation a thrombotic pannus was found extending from the sewing cuff into the orifice of both inflow and outflow sides of the valve. Both leaflets of the prosthesis were found fixed in a closed position.

Three years later (December 2003), the patient underwent her third open heart to deal with a malfunctioning prosthetic aortic valve. During operation a well formed circular pannus was found at the left ventricular aspect beneath the prosthesis, not extending to the orifice. Movement of valve leaflets was intact. The pannus was excised without replacing the aortic prosthesis.

The patient was followed up regularly. She was adequately anticoagulated. Repeated echocardiography showed a well functioning prosthetic valve.

On recent admission, the dysfunctional prosthetic aortic valve was exposed. An exactly similar pathology to the previous operation was encountered. A well circumscribed fibrotic pannus was found few mm beneath the prosthesis causing left ventricular outflow tract obstruction. Complete excision of the pannus was performed leaving the prosthetic valve intact. Postoperatively, the patient developed temporary left sided hemiparesis from which she recovered completely.

Prosthetic valve dysfunction in our case represents two different mechanisms causing malfunction. A thrombotic pannus formation affecting the mitral prosthesis and a recurrent pannus formation at the aortic position. The exact etiology of the recurrent pannus formation which necessitated repeated operation in our patient is unclear.

A previous study by Sakamoto and colleagues [2], found that the use of small bileaflet valves can promote pannus formation as well as the turbulent transvalvular blood flow across tilting disc valves.A high incidence of pannus formation at aortic position was found to be associated with St. Jude Medical valves (SJM). Ayogi and colleagues [9] suggested that pannus formation among these valves is initiated from the protruding design of the pivot guard system of SJM valves.

In the present case, no tilting valve was used, the size of the prosthetic aortic valve was appropriate and the formed pannus was found away from prosthetic valve.

It is interesting to note that most of the previously reported cases of pannus formation at aortic position were females including our case.

Dysfunctional prosthetic valves requires urgent diagnosis and surgical intervention. In the majority of cases, TTE can provide diagnosis and hemodynamic data. When combined with cine- radiography valuable information about leaflet movement can be provided [7]. The use of multidetector-row computed tomography is a useful technique used to evaluate dysfunctional prosthetic valves. When combined with cineradiography, both can demonstrate any abnormal small tissue extending from the left ventricular septum into the prosthetic aortic valve [10]. In technically inadequate or borderline cases, transoesophaged echocardiography (TEE) and more recently, the use of three-dimensional (3D) TEE was shown to be effective in diagnosis and visualization of leaflet immobility [1,11].

The diagnosis of valve dysfunction in our case was obtained by using TTE. However, during the second emergency operation, cine- radiography alone was sufficient to diagnose leaflet immobility.

In the majority of previously reported cases, an excision of the pannus together with valve re-replacement was performed. In few cases [7,8] in which the pannus was not encroaching on the prosthetic valve as in the present case, the valve was spared.

Several factors determined the decision to preserve the valve in our case, namely the location of the pannus which was localized and situated away from the prosthesis without interfering with its function, also the difficulty in exposing the prosthetic valve and the presence of extensive adhesions and calcification along the aortic wall. All these factors would have made surgery more complicated if the valve was re-replaced.

Excision of the pannus without re-replacement of the valve is an effective technique. The time lapse between excision of the pannus the first time and the present operation was almost 8 years. Postoperative TTE showed a well functioning aortic prosthesis with minimal gradient. Nevertheless, sparing the prosthesis did not prevent recurrent pannus formation.

In conclusion, patients presented with localized pannus not encroaching on the prosthetic valve can be successfully managed by excision of the pannus with sparing the valve. Several points need to be addressed, whether pannus formation is simply an exaggeration of an inflammatory healing process, is female gender a predisposing factor and whether sparing of the prosthetic valve can initiate future recurrence of pannus formation.
